# Encouragement of Enzyme Reaction Utilizing Heat Generation from Ferromagnetic Particles Subjected to an AC Magnetic Field

**DOI:** 10.1371/journal.pone.0127673

**Published:** 2015-05-19

**Authors:** Masashi Suzuki, Atsushi Aki, Toru Mizuki, Toru Maekawa, Ron Usami, Hisao Morimoto

**Affiliations:** 1 Graduate School of Interdisciplinary New Science, Toyo University, Kawagoe, Saitama, Japan; 2 Bio-Nano Electronics Research Center, Toyo University, Kawagoe, Saitama, Japan; Argonne National Laboratory, UNITED STATES

## Abstract

We propose a method of activating an enzyme utilizing heat generation from ferromagnetic particles under an ac magnetic field. We immobilize α-amylase on the surface of ferromagnetic particles and analyze its activity. We find that when α-amylase/ferromagnetic particle hybrids, that is, ferromagnetic particles, on which α-amylase molecules are immobilized, are subjected to an ac magnetic field, the particles generate heat and as a result, α-amylase on the particles is heated up and activated. We next prepare a solution, in which α-amylase/ferromagnetic particle hybrids and free, nonimmobilized chitinase are dispersed, and analyze their activities. We find that when the solution is subjected to an ac magnetic field, the activity of α-amylase immobilized on the particles increases, whereas that of free chitinase hardly changes; in other words, only α-amylase immobilized on the particles is selectively activated due to heat generation from the particles.

## Introduction

Enzymes are catalysts for chemical reactions in living organisms and are widely used in various areas such as biology, medicine, food industry, agriculture and so on. It is known that when enzymes are immobilized on appropriate carriers, their activities are stabilized even under high pH or high temperature conditions [1–6]. Magnetic particles are one of promising carriers for enzymes since enzymes immobilized on magnetic particles can easily be separated from a reaction solution using a magnet and they can be used repeatedly [7–13]. Such improvement in the reusability of enzymes contributes to reducing the cost of many industrial enzymatic processes. Another advantage of magnetic particles is that it is possible to control their motion and position using an alternating magnetic field or a gradient magnetic field and several methods of manipulating magnetic particles have been proposed in recent years [14–18]. The dynamics control and positioning of enzymes immobilized on magnetic particles in micro total analysis systems (μ-TAS) or micro reactors can be quite easily carried out using such manipulation methods. Recently, the activities of α-amylase, lipase and chitinase immobilized on superparamagnetic particles were investigated and it was reported that the activities of these enzymes on magnetic particles increased under relatively low frequency rotational magnetic fields, which was attributed to the rotational motion of the enzyme/particle hybrids induced by the external fields [19,20]. Ferromagnetic particles possess remanent magnetization and show magnetic hysteresis in contrast to superparamagnetic particles, in which case magnetization is induced only when the particles are subjected to an external magnetic field, and it is known that ferromagnetic particles generate heat under a high frequency ac magnetic field caused by magnetic hysteresis [21–23]. Skumiel et al. [24] investigated the heat generation of magnetic particles, the size of which was ranging from 1 to 5 μm, and reported that eddy currents also played a significant role in the heat generation of such micron-sized particles. If an enzyme is immobilized on ferromagnetic particles and the enzyme/ferromagnetic particle hybrids are subjected to a high frequency alternating magnetic field, the particles generate heat caused by magnetic hysteresis and eddy currents, and as a result, the activity of the enzyme immobilized on the particles may increase. Although the activities of enzymes immobilized in polymer gels, which contained ferromagnetic particles, under an alternating magnetic field were investigated [25,26], these studies mainly focused on the effect of deswelling-swelling of the gels induced by heat generation from the ferromagnetic particles on the activity. Klyachko et al. [27] investigated the effect of an ac magnetic field on the activities of enzymes immobilized on copolymer-magnetic nanoparticle aggregates. They reported that the activities of the enzymes were reduced under an ac magnetic field, which was attributed to conformational changes in the enzymes induced by realignment of magnetic nanoparticles rather than the heat generation of the particles, and to our knowledge, the effect of heat dissipation from ferromagnetic particles subjected to an alternating magnetic field on the activity of an enzyme immobilized on the particles has not yet been investigated in detail.

In this study, we immobilize α-amylase on ferromagnetic particles and investigate the enzyme reaction under a high frequency ac magnetic field. We show that when a high frequency ac magnetic field is applied to the α-amylase/ferromagnetic particle hybrids, the activity of immobilized α-amylase increases caused by heat generation from the particles. We estimate the average surface temperature of ferromagnetic particles under an ac magnetic field comparing the activity increase of α-amylase immobilized on particles under an ac magnetic field with the temperature dependence of the activity of immobilized α-amylase in the absence of a magnetic field. To make clear the effect of heat generation from ferromagnetic particles on free enzyme around the particles, we prepare a solution, in which α-amylase/ferromagnetic particle hybrids and free, nonimmobilized chitinase are dispersed, and analyze their activities. We show that only α-amylase immobilized on the particles is selectively heated up and activated with no influence on the activity of free chitinase under an ac magnetic field.

## Materials and Methods

### Sample Preparation

**Fig 1:**
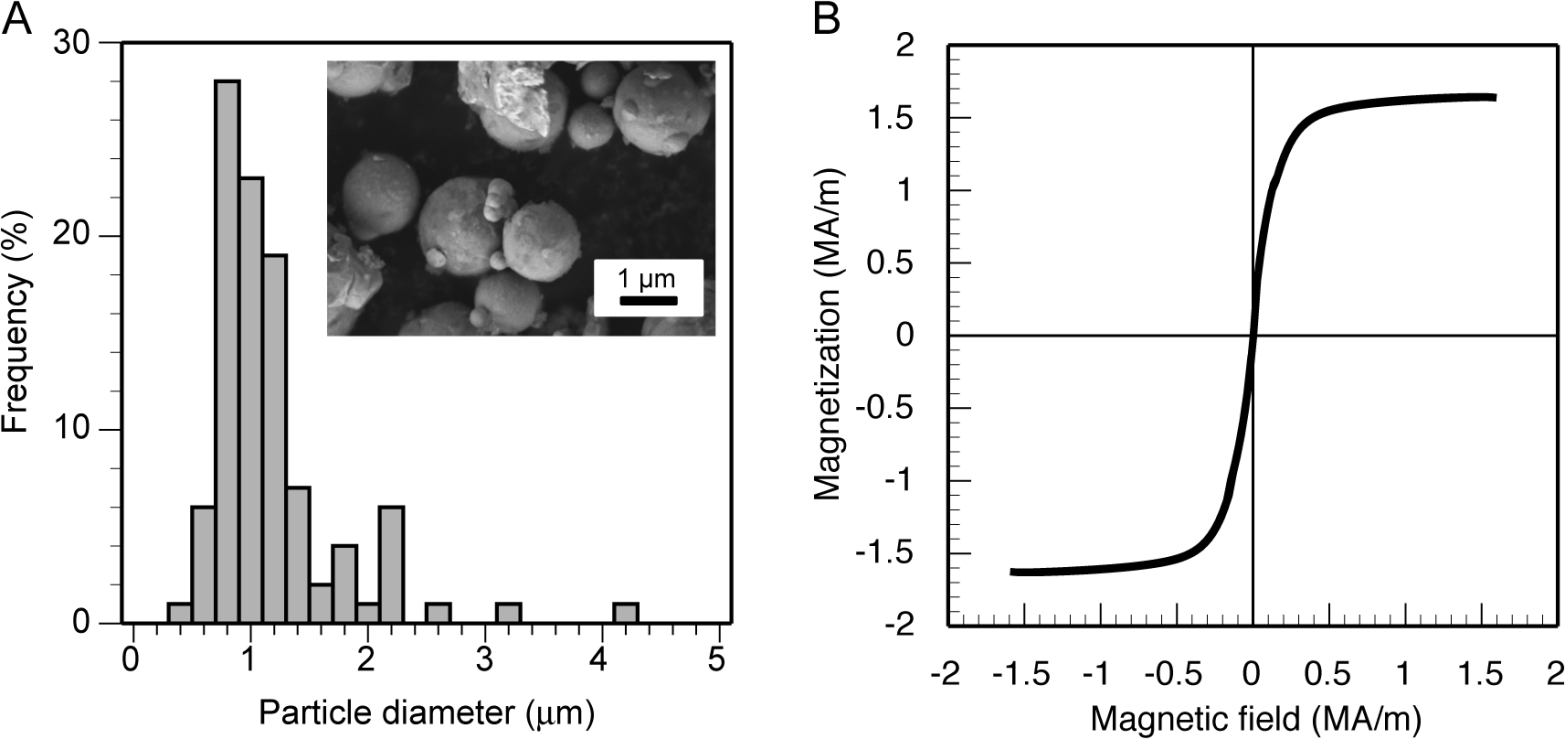
Ferromagnetic iron particles. (A) SEM image and size distribution of particles. The average diameter of particles is 1.1 ± 0.5 μm. (B) Hysteresis loop of particles.

α-amylase extracted from *Bacillus licheniformis* (E.C.3.2.1.1 type X2-A) and chitinase extracted from *Trichoderma viride* (E.C.3.2.1.14) were purchased from Sigma-Ardrich. Ferromagnetic particles used in this study (Spherical Ferromagnetic Iron Powder, Catalog No. 19844–1, Polysciences Inc.) were composed of iron and the surface of the particles was not modified with any molecules. We observed the particles using a scanning electron microscopy (SEM, JSM-7400F, JEOL) and an optical microscopy (TE2000-U, Nikon Corp.). The typical SEM image of particles and the particle size distribution obtained from the optical microscope images of particles are shown in Fig 1A. The average diameter of particles was estimated as 1.1 ± 0.5 μm. We measured the magnetic properties of the particles using a vibrating sample magnetometer (7407, LakeShore Cryotronics Inc.). The measured saturation magnetization was 1.64 MA/m. The remanent magnetization and the coercivity, which were estimated from the hysteresis loop obtained when the maximal intensity of the external magnetic field was set at 1.6 MA/m (see Fig 1B), were, respectively, 0.03 MA/m and 2.30 kA/m. We immobilized α-amylase on the surface of ferromagnetic particles as follows. First, 1 mg of ferromagnetic particles was added to 400 μL of α-amylase solution (19.8 μg/mL) and the mixture solution was left for 16 h at 4°C. After collecting the ferromagnetic particles using a magnet, the supernatant was removed and then the collected particles were redispersed into sterilized water. The above washing process was repeated three times to remove α-amylase molecules remaining in the solution, after which the volume fraction of the particles was set at 3.2 × 10^−2^% by adjusting the volume of a solvent fluid (water) relative to the total volume of the particles calculated from the total mass of the particles in the solution (1 mg). We measured the absorbance of 280 nm photons by the supernatant removed in the above washing procedure using a spectrophotometer (DU730, Beckman Coulter Inc.) and estimated the concentration of α-amylase in the supernatant from the dependence of the absorbance of 280 nm photons by aqueous solution of α-amylase on the concentration of α-amylase we measured. Note that the absorbance was proportional to the concentration in the concentration range we analyzed (0.01–0.2 g/L). We estimated the total number of α-amylase molecules contained in the supernatants removed in the washing procedure, from which the average number of α-amylase molecules immobilized on each ferromagnetic particle was calculated to be about 7.3 × 10^4^. Here, it should be noted that as the washing of the particles was repeated, the concentration of α-amylase in the removed supernatant estimated from the absorbance measurement was decreased and in the third washing treatment, the removed supernatant contained no detectable amount of α-amylase molecules, which means that there was no or a negligible amount of free α-amylase molecules in the particle solution after the washing procedure. We measured the magnetic properties of the α-amylase/ferromagnetic particle hybrids and confirmed that there was no significant difference in the magnetic properties between the original ferromagnetic particles and the hybrids since the ratio of the total mass of α-amylase immobilized on each particle to the mass of a particle was very low (0.14%).

Starch solution [0.2% starch dissolved in 100 mM citrate buffer (pH 6.0)] and para-nitrophenyl-N-acetyl-β-D-glucosaminide (pNP-NAG) solution [2 μM pNP-NAG dissolved in 100 mM phosphate buffer (pH 7.0)] were used as substrate solutions for α-amylase and chitinase respectively.

### Experimental System and Procedure

**Fig 2:**
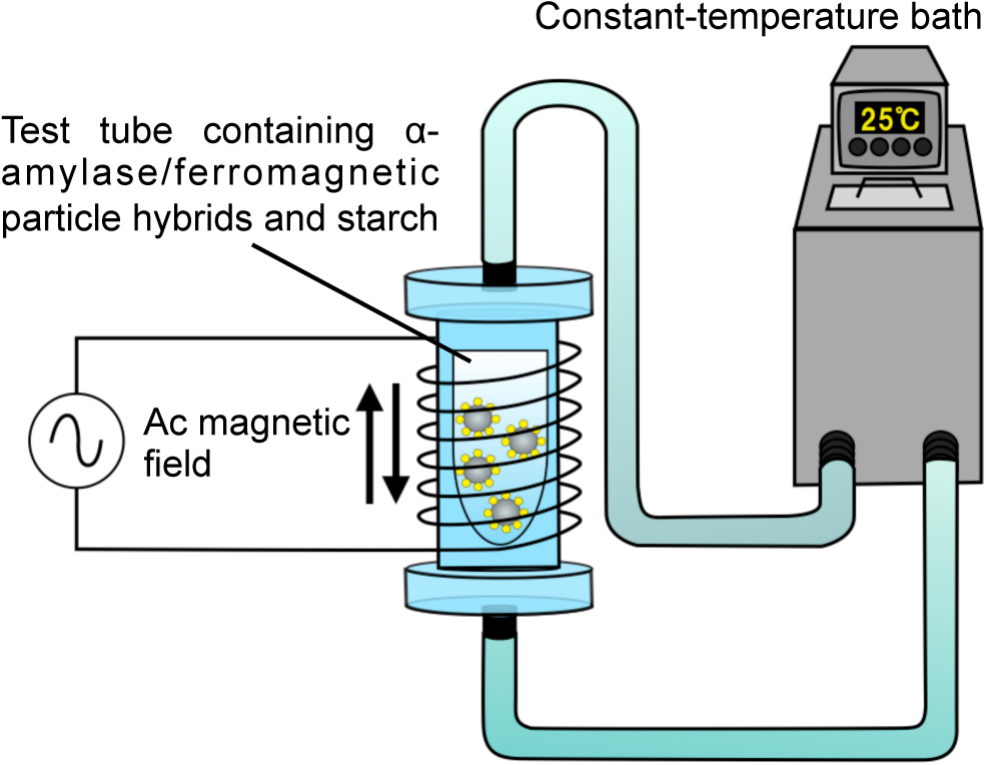
Schematic representation of an experimental system. A solution, in which α-amylase/ferromagnetic particle hybrids are dispersed, and starch solution are mixed in a test tube, which is placed in a cylindrical container filled with circulating water, the temperature of which is regulated at 25 °C, from a constant-temperature bath. Immediately after mixing the solutions, an ac magnetic field generated by a coil and an RF power supply is applied to the mixture solution. The enzyme reaction is carried out for 10 min and the activity of α-amylase is estimated by starch-iodine color reaction.

We analyzed the effect of heat dissipation from ferromagnetic particles under an ac magnetic field on the activity of α-amylase immobilized on the particles using an experimental system shown in Fig 2. 40 μL of α-amylase/ferromagnetic particle hybrid-dispersed solution and 500 μL of starch solution were mixed in a test tube, which was placed in a cylindrical container filled with circulating water, the temperature of which was regulated at 25°C, from a constant-temperature bath (LTB-400, AS ONE CO.). Immediately after mixing the solutions, an ac magnetic field of 0.34 MHz was applied to the mixture solution using a coil and a radio frequency (RF) power supply (LI8310, AMERITHERM Inc.). The amplitude of the ac magnetic field was changed from 15 to 30 kA/m. The enzyme reaction was terminated by mixing 500 μL of HCl (0.2 N) at 10 min after the application of the ac magnetic field and then the activity of α-amylase immobilized on ferromagnetic particles was evaluated by starch-iodine color reaction. We added 50 μL of an aqueous solution containing I_2_ (0.02%) and KI (0.2%) to the reaction solution and measured the absorbance of 700 nm photons, from which we calculated the activity of α-amylase.

We also analyzed the dependence of the activity of α-amylase immobilized on ferromagnetic particles in the absence of a magnetic field on the temperature using the same experimental system. We estimated the average surface temperature of ferromagnetic particles, on which α-amylase was immobilized, under the ac magnetic field comparing the activity increase of the immobilized α-amylase under the ac magnetic field and the temperature dependence of the activity in the absence of a magnetic field.

We next mixed α-amylase/ferromagnetic particle hybrid-dispersed solution with chitinase solution, where chitinase molecules did not attach to the ferromagnetic particles after the mixing and therefore they freely dispersed in the mixture solution, and examined the effect of heat generation from the particles under an ac magnetic field on the activities of both immobilized α-amylase and free, nonimmobilized chitinase. 20 μL of α-amylase/ferromagnetic particle hybrid-dispersed solution, 100 μL of chitinase aqueous solution (0.1 mg/mL), the substrate solution for α-amylase (250 μL) and that for chitinase (250 μL) were mixed in a test tube and then an ac magnetic field of 30 kA/m, the frequency of which was 0.34 MHz, was applied to the mixture solution at 25°C in the same manner as the previous experiment. At 10 min after the application of the ac magnetic field, 200 μL of the reaction solution was mixed with 200 μL of HCl (0.2 N) to terminate the reactions and the activity of α-amylase was estimated. The other 200 μL of the reaction solution was also mixed with 200 μL of HCl (0.2 N) at the same time, and it was used for analyzing the activity of chitinase. The activity of chitinase was evaluated as follows. First, we added 200 μL of phosphate buffer (1 M, pH 7.0) to the reaction solution to maintain the pH near neutral and then measured the adsorption of 405 nm photons by para-nitrophenol produced by the enzyme reaction, from which the activity of chitinase was calculated.

## Results and Discussion

**Fig 3:**
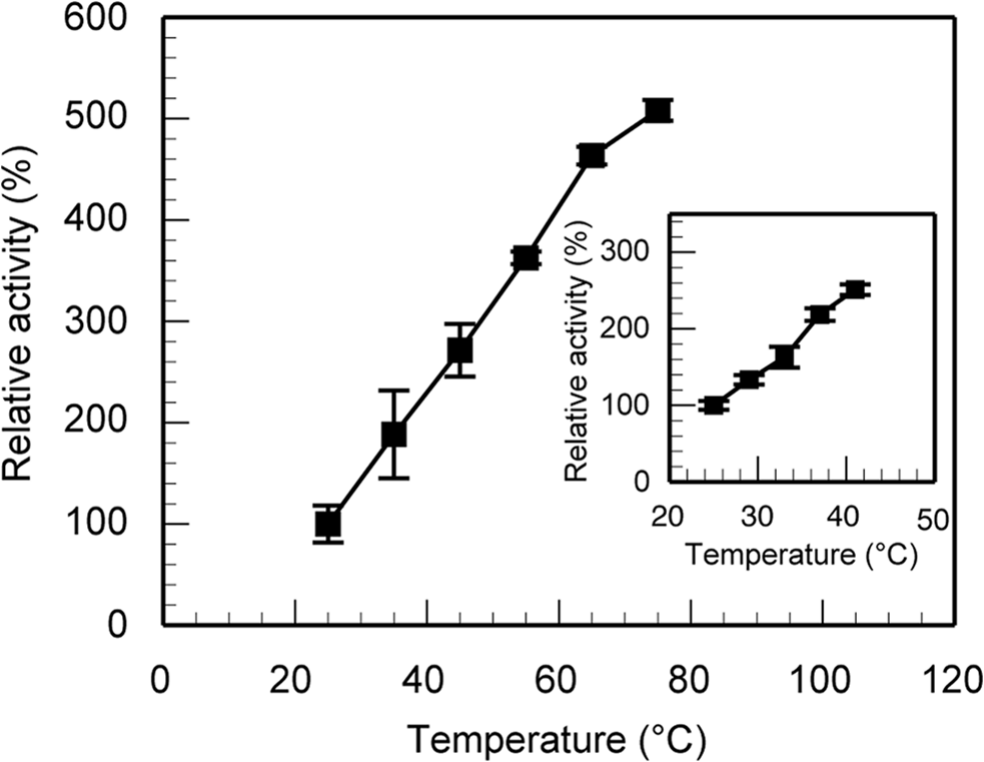
Dependence of the activity of α-amylase immobilized on ferromagnetic particles on the temperature. The ordinate axis represents the activity of α-amylase immobilized on ferromagnetic particles in the absence of a magnetic field, which is normalized by that measured at 25°C. The inset shows the detailed data between 25 and 41°C. The standard deviations are obtained from 3 independent experiments.

Ferromagnetic particles used in the present study were made of iron and their surface was not modified with any molecules as we mentioned in the previous section. Therefore, we suppose that α-amylase molecules were not bonded covalently to the particles but attached to the particles by electrostatic force, noting that the particles were negatively charged. Mizuki et al. [19] immobilized α-amylase on superparamagnetic particles, the surface of which was not modified with any molecules, via electrostatic force, noting that their particles are negatively charged and some domains in α-amylase are positively charged according to their numerical analysis (see Appendix S1 of [20]). In the present study, the particles we used were also negatively charged as mentioned above and therefore it is supposed that α-amylase molecules attached to the particles through their positively charged domains. To evaluate the effect of immobilization on the activity of α-amylase, we compared the activities of immobilized and free, nonimmobilized α-amylase in the absence of a magnetic field at 25°C. The activity of immobilized α-amylase was slightly decreased to 85 ± 16% compared to that of nonimmobilized one. However, according to the temperature dependence of the activity of immobilized α-amylase we measured (Fig 3), if we increase the temperature from 25°C to approximately 27°C, the activity of immobilized α-amylase becomes higher than that of nonimmobilized one at 25°C, which can easily be achieved by applying an ac magnetic field as we will show later. We next evaluated the effect of an ac magnetic field on the activities of enzymes used in the present study, that is, α-amylase and chitinase. Note that some enzymes are activated by applying a strong dc magnetic field or high dc gradient magnetic field, which is caused by the orientation or accumulation of the enzymes [28]. We estimated the activities of α-amylase and chitinase, which were not immobilized on ferromagnetic particles, under an ac magnetic field of 0.34 MHz, the amplitude of which was set at the maximal value in the present study (30 kA/m), and confirmed that there was no appreciable effect of the ac magnetic field on the activities of the enzymes since the amplitude of the ac magnetic field was so weak that no motion of enzyme molecules was induced.

**Fig 4:**
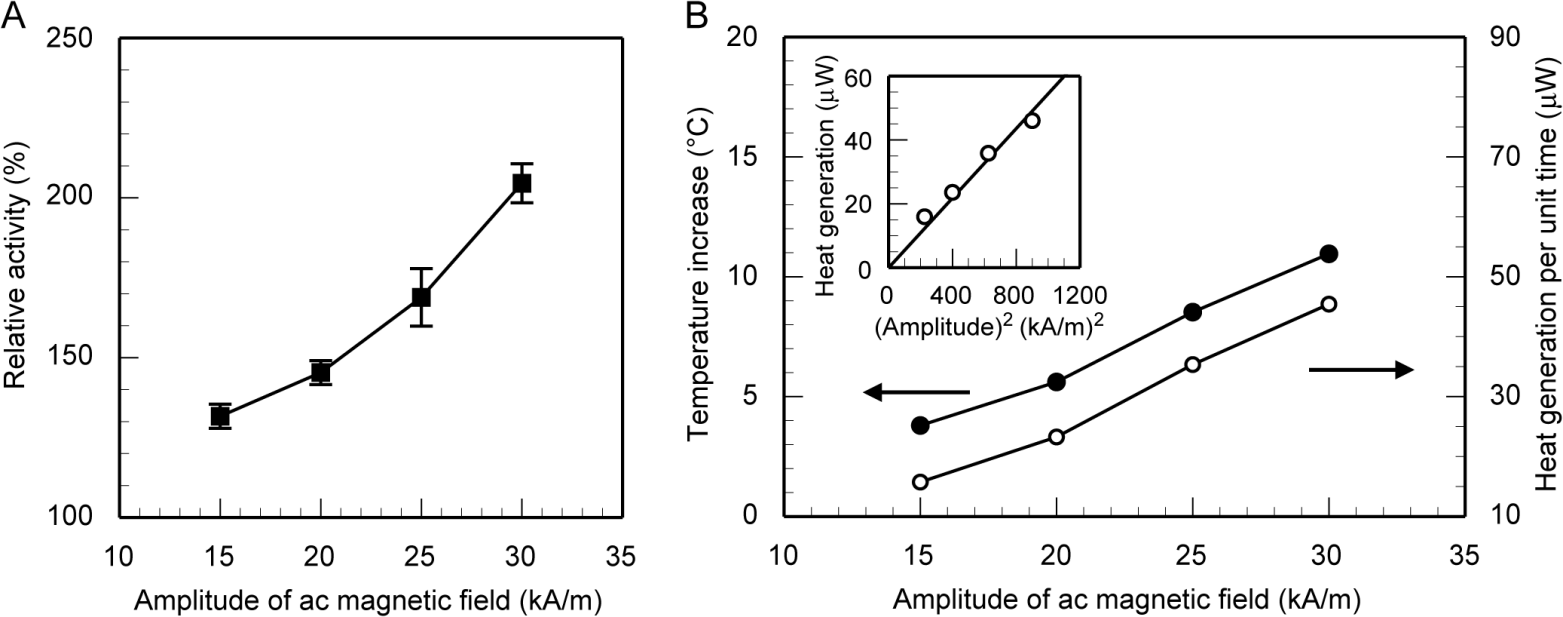
Activation of α-amylase immobilized on ferromagnetic particles under a high frequency ac magnetic field. (A) Dependence of the activity of α-amylase immobilized on the surface of ferromagnetic particles under an ac magnetic field of 0.34 MHz on the amplitude of the magnetic field. The ambient temperature is 25°C. The ordinate axis represents the activity of α-amylase immobilized on ferromagnetic particles under an ac magnetic field, which is normalized by that in the absence of a magnetic field. The standard deviations are obtained from 3 independent experiments. (B) Dependence of the surface temperature increase and the amount of heat generation per unit time of a ferromagnetic particle under an ac magnetic field of 0.34 MHz on the amplitude of the magnetic field. The surface temperature is estimated comparing the activity increase of α-amylase immobilized on ferromagnetic particles under the ac magnetic fields [see (A)] and the temperature dependence of the activity in the absence of a magnetic field (see Fig 3). The heat generation of a particle is calculated from the surface temperature and Eqs (3) and (4). The inset shows the plot of the heat generation versus the square of the amplitude.

The dependence of the activity of α-amylase immobilized on ferromagnetic particles under the ac magnetic field of 0.34 MHz on the amplitude of the field is shown in Fig 4A, where the activity is normalized by that in the absence of a magnetic field. α-amylase immobilized on ferromagnetic particles was activated due to heat generation from the particles under the ac magnetic fields and its activity increased with an increase in the amplitude of the ac magnetic field, which was caused by an increase in the amount of heat generated by the particles. In the present experiment, although the temperature of the reaction solution as a whole was kept at 25°C during the enzyme reaction, the ferromagnetic particles were locally heated up by the ac magnetic field and as a result, the enzyme on the particles was activated. In fact, no appreciable increase in the temperature of the reaction solution was detected since the volume fraction of the heat-dissipating ferromagnetic particles in the reaction solution was as small as 2.4 × 10^–3^%.

We estimated the average surface temperature of the ferromagnetic particles subjected to the ac magnetic fields. In the fluid region surrounding a particle, the heat should be transferred by conduction rather than convection since the characteristic length in the present case (i.e. the diameter of a particle) is extremely short and therefore the Rayleigh number should also be very small [29]. The relaxation time of heat conduction inside a particle and that in the fluid surrounding a particle were, respectively, 13 ns and 2.1 μs, both of which were much shorter than the reaction time in the present experiment (10 min). So we estimated the average surface temperature of the ferromagnetic particles comparing the activity of α-amylase immobilized on the particles under the ac magnetic fields (Fig 4A) and the temperature dependence of the activity in the absence of a magnetic field (Fig 3), supposing that the surface temperature reached its steady-state value immediately after the application of the field. The dependence of the amount of rise in the surface temperature on the amplitude of the field is shown in Fig 4B.

We next calculated the amount of heat generated by a ferromagnetic particle from the surface temperature estimated in the above. The volume fraction of the particles was very low in the present experiment and thus we assumed that the particles were thermally isolated from each other. Since the size of a ferromagnetic particle was as small as 1.1 μm and its thermal conductivity was relatively high (80 W/m K [30]), the temperature inside a particle can be assumed to be uniform, in which case the temporal evolution of the particles’ temperature, *T*, can be described as

$$\frac{dT}{dt}=-\frac{S_{p}}{\rho \;c_{p}V_{p}}q+\frac{W}{\rho \;c_{p}},$$ (1)

where *t*, *ρ*, *c*_*p*_, *S*_*p*_, *V*_*p*_, and *W* are, respectively, the time, the density, the specific heat, the surface area, the volume, and the amount of heat generation of a particle per unit time per unit volume. *q* is the average heat flux on the particles’ surface. We assumed that each ferromagnetic particle is spherical and that *q* is given by

$$q=\frac{\lambda_{f}}{a}\left(T-T_{f}\right),$$ (2)

where, *λ*_*f*_, *T*_*f*_, and *a* are, respectively, the thermal conductivity and the temperature of the surrounding fluid and the radius of a particle. The particles’ temperature as a function of the time obtained from Eqs (1) and (2), and the initial condition (when *t* = 0, *T* coincides with the temperature of the surrounding fluid, *T*_*f*_) is

$$T\left(t\right)=T_{f}+\frac{n}{m}\left\{1-\exp\left(-mt\right)\right\},$$ (3)

where the coefficients *m* and *n* are defined as follows

$$m\equiv \frac{3\lambda_{f}}{\rho \;c_{p}a^{2}},\;n\equiv \frac{W}{\rho \;c_{p}}.$$ (4)

Note that *T*_*f*_ corresponds to the average temperature of the reaction solution (25°C) in the present experiment. Using Eqs (3) and (4), and the steady-state particle’s surface temperature estimated in the above, we calculated the amount of heat generation of a particle per unit time under the ac magnetic fields, where the thermal conductivity of water (0.6 W/m K [30]), the density of iron (7874 kg/m^3^ [31]) and the specific heat of iron (449 J/kg K [30]) were respectively used for *λ*_*f*_, *ρ*, and *c*_*p*_ in Eq (4). The dependence of the heat generation on the amplitude of the field is also shown in Fig 4B. The heat generation was approximately proportional to the square of the amplitude (see the inset of Fig 4B). The specific absorption rate (SAR), which is defined as the heat power released per gram of magnetic material [32], can be obtained by dividing the amount of heat generation of a particle per unit time by the mass of a particle in grams. The SAR value calculated was ranging from 2.9 to 8.3 MW/g, which was much higher than those of magnetic nanosized particles reported in [32–34]. However, Baraban et al. [35] created magnetic Janus particles of 3 μm and the amount of heat generation of a particle per unit time was estimated to be 6 μW, which gives a high SAR value similar to the present calculation results. Furthermore, recent studies have reported significant temperature increase of magnetic particles subjected to ac magnetic fields, which also suggests high SAR values [36,37].

**Fig 5:**
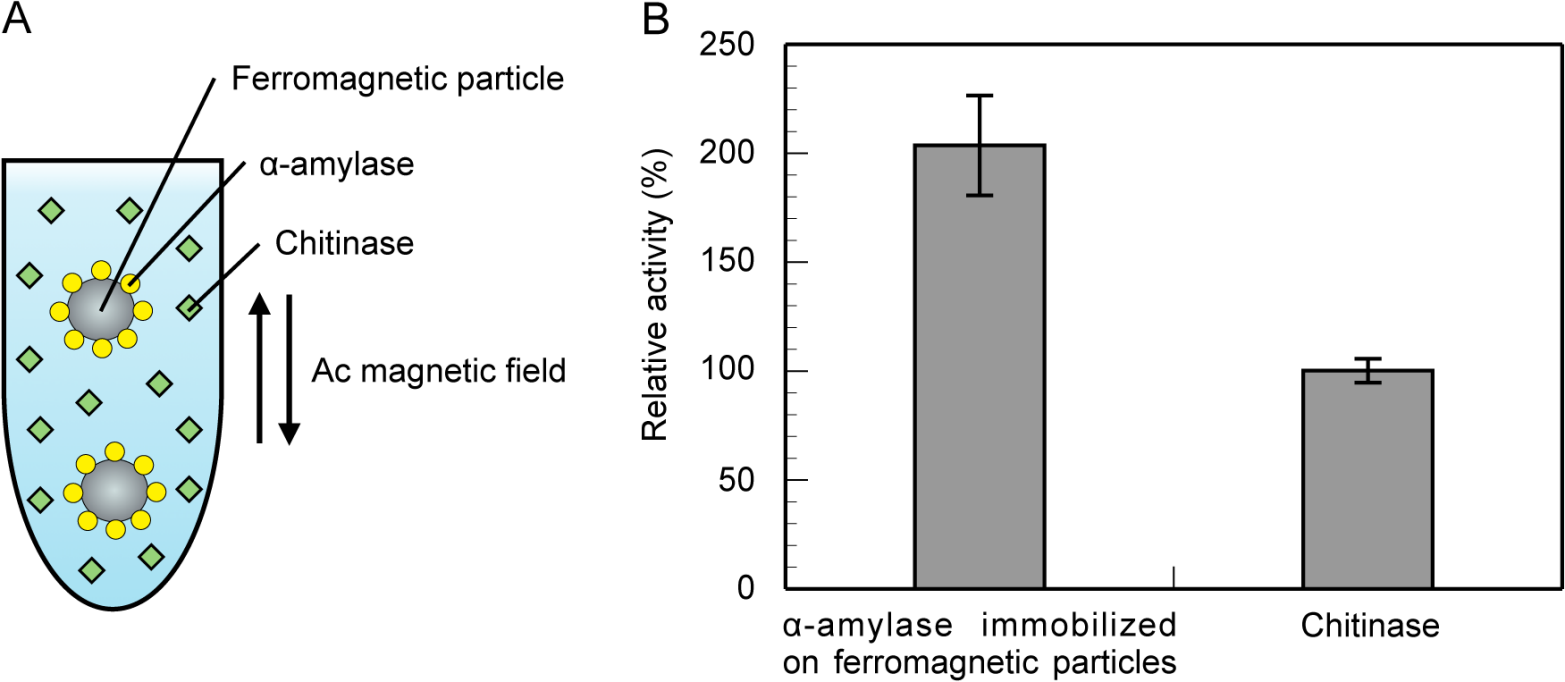
Activities of α-amylase immobilized on ferromagnetic particles and free chitinase under an ac magnetic field. (A) Schematic representation of the experiment. We prepare a solution, in which α-amylase/ferromagnetic particle hybrids and free, nonimmobilized chitinase are dispersed, and examine the effect of heat generation from the particles under an ac magnetic field on the activities of both immobilized α-amylase and nonimmobilized chitinase. (B) Activities of α-amylase immobilized on the surface of ferromagnetic particles and free chitinase under the ac magnetic field, the amplitude and frequency of which are, respectively, 30 kA/m and 0.34 MHz. The activity of each enzyme shown in the graph is normalized by that in the absence of a magnetic field. The standard deviations are obtained from 3 independent experiments.

Finally, we prepared a solution, in which α-amylase/ferromagnetic particle hybrids and free, nonimmobilized chitinase were dispersed, and analyzed the effect of heat generation from the particles under an ac magnetic field on the activities of both immobilized α-amylase and nonimmobilized chitinase (see Fig 5A). Fig 5B shows the relative activities of α-amylase immobilized on ferromagnetic particles and free chitinase under the ac magnetic field of 30 kA/m and 0.34 MHz, where the activity of each enzyme is normalized by that in the absence of a magnetic field. Under the ac magnetic field, the activity of α-amylase immobilized on ferromagnetic particles increased to about 204%, whereas that of chitinase hardly changed, which means that only α-amylase on the particles was selectively heated up and activated due to heat dissipation from the particles. The steady-state temperature distribution around a spherical particle obtained by solving the heat conduction equation is *T*(*r*) = *a*(*T*_*s*_−*T*_*f*_) / *r* + *T*_*f*_, where *r*, *a*, *T*_*s*_, and *T*_*f*_ are, respectively, the distance from the center of a particle, the radius of a particle, the surface temperature of a particle and the ambient temperature, which coincides with *T*(∞). The amount of rise in the average surface temperature of ferromagnetic particles subjected to the ac magnetic field, the amplitude and frequency of which were, respectively, 30 kA/m and 0.34 MHz, was estimated to be 11°C from the previous experimental result. In the case of *T*_*s*_−*T*_*f*_ = 11°C, the temperature increase, *T*(*r*)−*T*_*f*_, is higher than 1°C when *r* < 6.0 μm, in which region the activity of chitinase normalized by that at the ambient temperature (25°C) is ranging from 104 to 131% according to the temperature dependence of the activity we measured. However, the volume fraction of such regions was as low as 1.3% and therefore, there was no appreciable effect of heat generation from the particles on the activity of free chitinase under the present experimental conditions. This experimental result suggests that an enzyme immobilized on ferromagnetic particles can be activated by applying an ac magnetic field without any effect on the other molecules around the particles if we appropriately choose the experimental parameters, that is, the frequency, the amplitude and the application time of the ac magnetic field, and the volume fraction of enzyme/ferromagnetic particle hybrids.

In summary, we immobilized an enzyme on the surface of ferromagnetic particles and successfully activated the immobilized enzyme utilizing heat generation from the particles under an ac magnetic field. In the present activation method, enzyme molecules are attached to heat sources, that is, ferromagnetic particles, and therefore the enzymes may be heated and activated rapidly under an ac magnetic field. Since a small region surrounding a ferromagnetic particle is locally heated by an ac magnetic field, we can carry out enzyme reactions with a high reaction rate even under low ambient temperature conditions, which minimizes undesirable chemical reactions that can occur at high temperatures. The above features are clearly useful for enzyme reactions, which need to be carried out at lower temperatures compared to the optimal temperatures of enzymes for several reasons [38–43]. For instance, some enzyme reactions utilized in food industry are carried out at low temperature to prevent modification of their heat-sensitive substrates and products [39–42]. DNA ligation is also usually carried out at low temperature compared to the optimal temperature of DNA ligase to allow the ends of DNA fragments to be associated with each other efficiently [43]. If we use enzyme/ferromagnetic particle hybrids, such enzyme reactions under low temperature conditions can be encouraged by applying an ac magnetic field. Furthermore, since magnetic particles can be manipulated using an external magnetic field [14–18], the manipulation and reaction control of enzymes in micro total analysis systems (μ-TASs) can be carried out utilizing enzyme/ferromagnetic particle hybrids. So we are convinced that our activation method may also make a great contribution to both bio-nano science and technology. We will be investigating the effect of the experimental parameters, that is, the frequency, the amplitude and the application time of the ac magnetic field, and the volume fraction of enzyme/ferromagnetic particle hybrids, on the enzymes’ activity in detail. We will also be investigating the effect of heat generation from ferromagnetic particles under an ac magnetic field on the activities of other types of enzymes such as DNA ligase, DNA polymerase, invertase and so on.

## References

[1] Chien L-J, Lee C-K. Biosilicification of dual-fusion enzyme immobilized on magnetic nanoparticle. Biotechnol Bioeng. 2008; 100(2): 223–230. 18078291 10.1002/bit.21750

[2] Asuri P, Bale SS, Pangule RC, Shah DA, Kane RS, Dordick JS. Structure, Function, and Stability of Enzymes Covalently Attached to Single-Walled Carbon Nanotubes. Langmuir. 2007; 23(24): 12318–12321. 17944500 10.1021/la702091c

[3] Tsang SC, Yu CH, Gao X, Tam K. Silica-Encapsulated Nanomagnetic Particle as a New Recoverable Biocatalyst Carrier. J Phys Chem B. 2006; 110(34): 16914–16922. 16927981 10.1021/jp062275s

[4] Deng HT, Xu ZK, Huang XJ, Wu J, Seta P. Adsorption and Activity of *Candida rugosa* Lipase on Polypropylene Hollow Fiber Membrane Modified with Phospholipid Analogous Polymers. Langmuir. 2004; 20(23): 10168–10173. 15518509 10.1021/la0484624

[5] Mukhopadhyay K, Phadtare S, Vinod VP, Kumar A, Rao M, Chaudhari RV, et al Gold Nanoparticles Assembled on Amine-Functionalized Na-Y Zeolite: A Biocompatible Surface for Enzyme Immobilization. Langmuir. 2003; 19(9): 3858–3863.

[6] Matsumoto M, Ohashi K. Effect of immobilization on thermostability of lipase from *Candida rugosa* . Biochem Eng J. 2003; 14(1): 75–77.

[7] Kalkan NA, Aksoy S, Aksoy EA, Hasirci N. Preparation of Chitosan-Coated Magnetite Nanoparticles and Application for Immobilization of Laccase. J Appl Polym Sci. 2012; 123(2): 707–716.

[8] Uygun DA, Ozturk N, Akgol S, Denizli A. Novel Magnetic Nanoparticles for the Hydrolysis of Starch with *Bacillus licheniformis* α-Amylase. J Appl Polym Sci. 2012; 123(5): 2574–2581.

[9] Kuo C-H, Liu Y-C, Chang C-MJ, Chen J-H, Chang C, Shieh C-J. Optimum conditions for lipase immobilization on chitosan-coated Fe_3_O_4_ nanoparticles. Carbohydr Polym. 2012; 87(4): 2538–2545.

[10] Maciel JC, Andrad PL, Neri DFM, Carvalho LB Jr, Cardoso CA, Calazans GMT, et al Preparation and characterization of magnetic levan particles as matrix for trypsin immobilization. J Magn Magn Mater. 2012; 324(7): 1312–1316.

[11] Ranjbakhsh E, Bordbar AK, Abbasi M, Khosropour AR, Shams E. Enhancement of stability and catalytic activity of immobilized lipase on silica-coated modified magnetite nanoparticles. Chem Eng J. 2012; 179: 272–276.

[12] Park HJ, McConnell JT, Boddohi S, Kipper MJ, Johnson PA. Synthesis and characterization of enzyme-magnetic nanoparticle complexes: effect of size on activity and recovery. Colloids Surf B Biointerfaces. 2011; 83(2): 198–203. 10.1016/j.colsurfb.2010.11.006 21176875

[13] Matsune H, Jogasaki H, Date M, Takenaka S, Kishida M. One-pot Synthesis and Characterization of Laccase-entrapped Magnetic Nanobeads. Chem Lett. 2006; 35(12): 1356–1357.

[14] Peyer KE, Zhang L, Nelson BJ. Bio-inspired magnetic swimming microrobots for biomedical applications. Nanoscale. 2013; 5(4): 1259–1272. 10.1039/c2nr32554c 23165991

[15] Sing CE, Schmid L, Schneider MF, Franke T, Alexander-Katz A. Controlled surface-induced flows from the motion of self-assembled colloidal walkers. Proc Natl Acad Sci U S A. 2010; 107(2): 535–540. 10.1073/pnas.0906489107 20080716 PMC2818967

[16] Aki A, Ito O, Morimoto H, Nagaoka Y, Nakajima Y, Mizuki T, et al Capture of nonmagnetic particles and living cells using a microelectromagnetic system. J Appl Phys. 2008; 104(9): 094509 10.1063/1.3010307

[17] Tierno P, Golestanian R, Pagonabarraga I, Sagues F. Magnetically Actuated Colloidal Microswimmers. J Phys Chem B. 2008; 112(51): 16525–16528. 10.1021/jp808354n 19367983

[18] Morimoto H, Ukai T, Nagaoka Y, Grobert N, Maekawa T. Tumbling motion of magnetic particles on a magnetic substrate induced by a rotational magnetic field. Phys Rev E. 2008; 78(2): 021403 10.1103/PhysRevE.78.021403 18850832

[19] Mizuki T, Watanabe N, Nagaoka Y, Fukushima T, Morimoto H, Usami R, et al Activity of an enzyme immobilized on superparamagnetic particles in a rotational magnetic field. Biochem Biophys Res Commun. 2010; 393(4): 779–782. 10.1016/j.bbrc.2010.02.081 20171160

[20] Mizuki T, Sawai M, Nagaoka Y, Morimoto H, Maekawa T. Activity of Lipase and Chitinase Immobilized on Superparamagnetic Particles in a Rotational Magnetic Field. Plos One. 2013; 8(6): e66528 10.1371/journal.pone.0066528 23799111 PMC3682989

[21] Mary APR, Narayanan TN, Sunny V, Sakthikumar D, Yoshida Y, Joy PA, et al Synthesis of Bio-Compatible SPION-based Aqueous Ferrofluids and Evaluation of RadioFrequency Power Loss for Magnetic Hyperthermia. Nanoscale Res Lett. 2010; 5(10): 1706–1711. 21076702 10.1007/s11671-010-9729-4 PMC2956030

[22] Fortin J-P, Wilhelm C, Servais J, Menager C, Bacri J-C, Gazeau F. Size-Sorted Anionic Iron Oxide Nanomagnets as Colloidal Mediators for Magnetic Hyperthermia. J Am Chem Soc. 2007; 129(9): 2628–2635. 17266310 10.1021/ja067457e

[23] Bae S, Lee SW, Takemura Y. Applications of NiFe_2_O_4_ nanoparticles for a hyperthermia agent in biomedicine. Appl Phys Lett. 2006; 89(25): 252503 10.1063/1.2420769

[24] Skumiel A, Kaczmarek-Klinowska M, Timko M, Molcan M, Rajnak M. Evaluation of Power Heat Losses in Multidomain Iron Particles Under the Influence of AC Magnetic Field in RF Range. Int J Thermophys. 2013; 34(4): 655–666.

[25] Kato N, Oishi A, Takahashi F. Enzyme reaction controlled by magnetic heating due to the hysteresis loss of γ-Fe_2_O_3_ in thermosensitive polymer gels immobilized β-galactosidase. Mater Sci Eng C. 1998; 6(4): 291–296.

[26] Takahashi F, Sakai Y, Mizutani Y. Immobilized Enzyme Reaction Controlled by Magnetic Heating: γ-Fe_2_O_3_-Loaded Thermosensitive Polymer Gels Consisting of *N*-Isopropylacrylamide and Acrylamide. J Ferment Bioeng. 1997; 83(2): 152–156.

[27] Klyachko NL, Sokolsky-Papkov M, Pothayee N, Efremova MV, Gulin DA, Pothayee N, et al Changing the Enzyme Reaction Rate in Magnetic Nanosuspensions by a Non-Heating Magnetic Field. Angew Chem Int Ed. 2012; 51(48): 12016–12019. 10.1002/anie.201205905 23081706 PMC3571765

[28] Haberditzl W. Enzyme Activity in High Magnetic Fields. Nature. 1967; 213: 72–73.

[29] Jayhooni SMH, Rahimpour MR. Effect of different types of nanofluids on free convection heat transfer around spherical mini-reactor. Superlattices Microstruct. 2013; 58: 205–217.

[30] Lide DR, editor. CRC Handbook of chemistry and physics 76th ed. Boca Raton: CRC Press; 1995.

[31] Coey JMD. Magnetism and magnetic materials Cambridge: Cambridge University Press; 2010.

[32] Ming M, Ya W, Jie Z, Yongkang S, Yu Z, Ning G. Size dependence of specific power absorption of Fe_3_O_4_ particles in AC magnetic field. J Magn Magn Mater. 2004; 268(1–2): 33–39.

[33] Baker I, Zeng Q, Li WD, Sullivan CR. Heat deposition in iron oxide and iron nanoparticles for localized hyperthermia. J Appl Phys. 2006; 99(8): 08H106 10.1063/1.2171960

[34] Guardia P, Di Corato R, Lartigue L, Wilhelm C, Espinosa A, Garcia-Hernandez M, et al Water-Soluble Iron Oxide Nanocubes with High Values of Specific Absorption Rate for Cancer Cell Hyperthermia Treatment. Acs Nano. 2012; 6(4): 3080–3091. 10.1021/nn2048137 22494015

[35] Baraban L, Streubel R, Makarov D, Han L, Karnaushenko D, Schmidt OG, et al Fuel-Free Locomotion of Janus Motors: Magnetically Induced Thermophoresis. Acs Nano. 2013; 7(2): 1360–1367. 10.1021/nn305726m 23268780

[36] Riedinger A, Guardia P, Curcio A, Garcia MA, Cingolani R, Manna L, et al Subnanometer Local Temperature Probing and Remotely Controlled Drug Release Based on Azo-Functionalized Iron Oxide Nanoparticles. Nano Lett. 2013; 13(6): 2399–2406. 10.1021/nl400188q 23659603

[37] Dias JT, Moros M, del Pino P, Rivera S, Grazu V, de la Fuente JM. DNA as a Molecular Local Thermal Probe for the Analysis of Magnetic Hyperthermia. Angew Chem Int Ed. 2013; 52(44): 11526–11529. 10.1002/anie.201305835 24115553

[38] Shahsavarani H, Sugiyama M, Kaneko Y, Chuenchit B, Harashima S. Superior thermotolerance of *Saccharomyces cerevisiae* for efficient bioethanol fermentation can be achieved by overexpression of *RSP5* ubiquitin ligase. Biotechnol Adv. 2012; 30(6): 1289–1300. 10.1016/j.biotechadv.2011.09.002 21930195

[39] Groudieva T, Kambourova M, Yusef H, Royter M, Grote R, Trinks H, et al Diversity and cold-active hydrolytic enzymes of culturable bacteria associated with Arctic sea ice, Spitzbergen. Extremophiles. 2004; 8(6): 475–488. 15252724 10.1007/s00792-004-0409-0

[40] Nakagawa T, Fujimoto Y, Uchino M, Miyaji T, Takano K, Tomizuka N. Isolation and characterization of psycrophiles producing cold-active *β*-garactosidase. Lett Appl Microbiol. 2003; 37(2): 154–157. 12859659 10.1046/j.1472-765x.2003.01369.x

[41] Gerday C, Aittaleb M, Bentahir M, Chessa JP, Claverie P, Collins T, et al Cold-adapted enzymes: from fundamentals to biotechnology. Trends Biotechnol. 2000; 18(3): 103–107. 10675897 10.1016/s0167-7799(99)01413-4

[42] Marshall CJ. Cold-adapted enzymes. Trends Biotechnol. 1997; 15(9): 359–364. 9293034 10.1016/S0167-7799(97)01086-X

[43] Lund AH, Duch M, Pedersen FS. Increased cloning efficiency by temperature-cycle ligation. Nucleic Acids Res. 1996; 24(4): 800–801. 8604328 10.1093/nar/24.4.800 PMC145669

